# Use of Biostimulants for Organic Apple Production: Effects on Tree Growth, Yield, and Fruit Quality at Harvest and During Storage

**DOI:** 10.3389/fpls.2018.01342

**Published:** 2018-09-20

**Authors:** Sebastian Soppelsa, Markus Kelderer, Claudio Casera, Michele Bassi, Peter Robatscher, Carlo Andreotti

**Affiliations:** ^1^Faculty of Science and Technology, Free University of Bozen-Bolzano, Bolzano, Italy; ^2^Laimburg Research Centre, Vadena, Italy

**Keywords:** *Malus* × *domestica*, seaweed extract, photosynthesis, phenolic compounds, anthocyanins, physiological disorders, organic production

## Abstract

The experiment was conducted during two consecutive seasons (years 2016 and 2017) in an organic apple orchard of the cultivar Jonathan. Several biostimulants were tested (10 in total), including humic acids, macro and micro seaweed extracts, alfalfa protein hydrolysate, amino acids alone or in combination with zinc, B-group vitamins, chitosan and a commercial product containing silicon. Treatments were performed at weekly intervals, starting from the end of May until mid-August. The macroseaweed extract was effective in stimulate tree growth potential in both years, as shown by a significantly larger leaf area (+20% as compared to control) and by an higher chlorophyll content and leaf photosynthetic rate in year 2016. As for the yield performances and apples quality traits at harvest (average fruit weight, soluble solids content, titratable acidity, and flesh firmness), they were generally affected by the different climatic conditions that characterized the two growing seasons (year 2017 being characterized by higher maximal and average temperatures and by limited rainfalls at the beginning of the season). Treatments with macroseaweed extract, B-group vitamins and alfalfa protein hydrolysate were able to significantly improve the intensity and extension of the red coloration of apples at harvest. Correspondingly, the anthocyanin content in the skin of apples treated with the same biostimulants resulted significantly higher than control, highlighting the potential influence of these substances on the synthesis of secondary metabolites in apple. The incidence of physiological disorders was also monitored during apple storage period. Amino acids plus zinc application was effective in reducing (more than 50%) the incidence of the “Jonathan spot,” the main post-harvest disorder for this cultivar.

## Introduction

Organic farming, including organic apple production, is generally characterized by lower crop yield as compared with conventional production systems mainly because of the limitation imposed on fertilization (no use of chemical fertilizers) and on plant defense (no use of pesticides) (Amarante et al., 2008; de Ponti et al., 2012; Seufert et al., 2012; Orsini et al., 2016). In order to reduce this gap of productivity, the organic agriculture sector is therefore constantly seeking for new agroecological practices to integrate in the management of the cultivation systems. Biostimulants are considered among the most innovative and promising solutions to improve sustainability and profitability of organic agriculture (Povero et al., 2016). Biostimulants are defined as “*any substance or microorganism applied to plants with the aim to enhance nutrition efficiency, abiotic stress tolerance and/or crop quality traits, regardless of its nutrients content*” (du Jardin, 2015). The main categories of plant biostimulants include natural substances such as humic and fulvic acids, protein hydrolysates, seaweed extracts (Battacharyya et al., 2015; Canellas et al., 2015; Colla et al., 2017), beneficial fungi (e.g., arbuscular mycorrhizal fungi and *Trichoderma* spp.) (Rouphael et al., 2015) and plant growth promoting rhizobacteria (Ruzzi and Aroca, 2015). Other substances (e.g., vitamins, chitosan and other biopolymers, inorganic compounds) can have biostimulant properties, but their classification within the group of biostimulants is still under consideration.

Organic farming can benefit from the use of biostimulants because these substances can enhance plant resilience to the nutrient limitation typical of this production system, therefore reducing the gap between organic and conventional yields (De Pascale et al., 2017). The increase of nutrient uptake and assimilation by biostimulant substances can follow different mechanisms. Biostimulants such as humic substances and protein hydrolysates can enhance nutrient availability by changing the physico-chemical properties of soils (i.e., increasing the cation exchange capacity of sandy soils) and by forming complexes with micronutrients more available to plants (Canellas et al., 2015; Colla et al., 2017). Moreover, the use of biostimulants (e.g., humic acids, protein hydrolysates, and seaweed extracts) can promote root growth and development, allowing a better soil exploration and consequently nutrient uptake (Battacharyya et al., 2015; Hernández-Herrera et al., 2016; Scaglia et al., 2016; Colla et al., 2017). The nutrient assimilation process can be also positively affected by biostimulants as shown by the increased activity of key enzymes (e.g., nitrate reductase) following the application of bioactive substances (protein hydrolysates and seaweed extracts) to roots and leaves (Schiavon et al., 2008; Ertani et al., 2009; Zhang et al., 2010). Despite the large and increasing number of publications dealing with biostimulants (Colla and Rouphael, 2015), scientific-based information on their optimal use, crop specificity, and interaction with the growing conditions is anyway still incomplete. Studies on the effect of biostimulants on the growth and yield potential of plants have been conducted primarily on vegetable crops. Rouphael et al. (2017) and Ertani et al. (2015) on tomato and pepper, respectively, demonstrated how protein hydrolysates were able to increase plant productivity, probably because of a stimulation of the plant primary metabolism trigged by signaling molecules (peptides, oligopeptides, and free amino acids) contained in the hydrolysate. This fostering effect on the primary metabolism is particularly significant when plants are under stress conditions, as demonstrated for tomato and spinach suffering of drought and treated with seaweed extracts (Xu and Leskovar, 2015; Goñi et al., 2018). Similar studies are more complex when conducted on woody plants, also due to the role played by nutrient reserves stored in the permanent woody structure of the tree for its growth metabolism. This could be one of the reasons explaining the current lack of solid evidences connecting the use of biostimulant compounds with the final growth and yield of fruit crops.

Biostimulants have been found active in promoting final crop quality and, more in detail, researches had highlighted the relevance of biostimulant applications for selected functional quality traits. The concentration of secondary metabolites such as phenols, flavonoids, and ascorbic acid were enhanced after the application of protein hydrolysates or seaweed extract in tomato (Rouphael et al., 2017), pepper (Ertani et al., 2015), onion, and potato (Lola-Luz et al., 2014). Giving the high antioxidant activity of phenolic compounds, the nutraceutical value of those vegetables was also improved. The physiological mechanism behind these results is the up-regulation of genes responsible for the secondary metabolism in plants treated with the biostimulants as demonstrated with the microarrays technique on tomato (Ertani et al., 2017). Color is another crop quality trait considered as very relevant because of its tight connection with the consumer-choice behavioral mechanism. In the case of apple fruits, color intensity and extension is largely related to the anthocyanin biosynthesis and accumulation in skin tissue (Liu et al., 2013). In order to promote apple final coloration at harvest, several cultivation techniques have been tested such as defoliation, reflective mulches in the inter-row space prior to harvest, overhead sprinkler irrigation, and fruit bagging (Whale et al., 2008; Blanke and Kunz, 2016). In addition, also the use of synthetic growth regulators (ethephon) to stimulate pigments biosynthesis can be an option, even though not allowed by the organic production system (Ubi, 2004). Selected biostimulants have been found able to improve final coloration of different fruit crops (Roussos et al., 2009; Portu et al., 2015; Blanke and Kunz, 2016) probably thanks to their capacity to modulate the activity of endogenous plant hormones (Wally et al., 2013), leading to an induction of the anthocyanin biosynthetic pathway at fruit skin level.

Biostimulants could also be considered for their implementation in the post-harvest management of fruits. Biostimulants containing mineral nutrients such as zinc and silicon might contribute with calcium to the strengthening of cell wall structure (Ferguson et al., 1999), therefore allowing the preservation of fruit quality attributes for longer period. This is of particular interest for the organic apple production system that is presently lacking of any useful means to manage apple physiological disorders during storage.

Our field experiment was conducted in an organic apple orchard located in the Alto-Adige/South Tyrol Province, northeast of Italy. This area is the major apple-growing district of Italy, accounting for approximately 65% of the total national and 10% of EU production (FAOSTAT, 2017, ISTAT, 2017). Pushed by the concern of the public opinion about the intensive use of pesticides in this area and by the favorable market conditions, the organic apple production sector has gained relevance constantly during the last years. Today around 10% of the apple orchard surface in Alto-Adige/South Tyrol is cultivated according to the organic protocol and about one third of all organic apples in Europe are harvested in this province (Alto-Adige/South Tyrol Province, 2017). To sustain further the growth and profitability of the organic apple sector, the implementation of new agroecological means, such as the biostimulant products, in the management of the organic horticultural systems is highly requested by the growers. The use of these new tools must follow anyway information deriving from scientifically sound research about their effects on plant physiological and biochemical responses. With this goal, this work aimed to investigate the effects of biostimulant applications on the growth, yield, and fruit quality of organically cultivated apple trees, belonging to the cv. Jonathan. Some of the selected substances were tested for the first time on apple crop and, to the best of our knowledge, this was the first study where the efficacy of several biostimulants was evaluated simultaneously and during two consecutive growing seasons. In addition, their effect was considered also during the storage period of fruits by measuring the incidence of the main post-harvest physiological disorder of “Jonathan” apples.

## Materials and Methods

### Experimental Site and Biostimulant Applications

**FIGURE 1:**
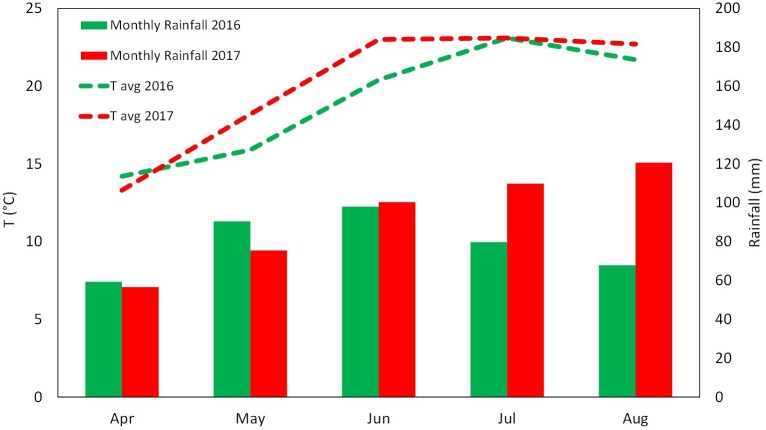
Meteorological conditions during the growing season 2016 and 2017 at the Laimburg Research Centre in Ora (Italy). Average temperature (dotted lines) and cumulated monthly rainfalls (columns).

The experiment was conducted over two growing seasons (years 2016 and 2017) in an experimental apple orchard located at the Laimburg Research Centre, in the municipality of Ora/Auer (46° 22′ North; 11° 17′ East; 237 m a.s.l.) in Alto Adige/South Tyrol, Italy. Meteorological conditions during the growing seasons (from April to August 2016 and 2017) are reported in **Figure 1**. The 8-year-old “Jonathan” apple trees (*Malus*×*domestica* Borkh. “Red Jonathan”) were grafted on M.9 rootstock, spaced 1.0 m × 3.0 m (3,333 trees ha^-1^), and trained to spindle system. The orchard received standard horticultural cares in accordance with the regulation governing organic production.

The experiment set up was organized as a completely randomized block design with four replications per treatment and five trees per replicate. To avoid any contamination between treatments, replicates on the same row were separated by an interval of 10 untreated trees, whereas a buffer row was used to separate plots on adjacent rows. Trees were selected according to their uniformity as for flowering and growth, by estimating the number of flowers per tree and measuring trunk circumference at 20 cm from the ground, respectively. The same set of trees was selected for the experiment in both the considered growing seasons. Biostimulant applications to the tree canopy started 40 days after full bloom (DAFB) at the end of May and were performed at weekly intervals until the end of August (1 week before harvest).

Details on the names, abbreviations, and physico-chemical characteristics of the utilized biostimulants, including the way of application are reported in **Table 1** and **Supplementary Table S1**. All treatments were performed with a total volume of 1,500 L ha^-1^; each tree was sprayed until the run-off point using a pulled sprayer under favorable weather forecast (no rainfalls expected in the following 24 h).

**Table 1:** Biostimulant characterization, properties, and mode of application.

Treatment	Active ingredients	Moisture (%)	Ash (%)	Density (kg dm^-3^)	Organic matter (%)	pH	Electrical conductivity (ds m^-1^)	Total organic carbon (% w w^-1^)	Total organic nitrogen (% w w^-1^)	Free amino acids (% w w^-1^)	Total amino acids (% w w^-1^)	Other charac-teristics	Concentration
CON	Water			
HAL	Humic acids	–	–	1.1	–	9.2	1.2	7.5	0.1	–	–	–	1.0 kg ha^-1^
APH	Alfalfa protein hydrolysate	70.0	7.0	1.2	23.0	5.5	1.6	–	–	1.5	5.1	More details in the annex	3.0 kg ha^-1^
SEA	Macroseaweed extract	84.0	1.5	1.0	14.5	4.5	0.4	3	≤0.1	–	–	From *A. nodosum*	4.0 kg ha^-1^
SPI	Microalga hydrolysate	–	–	1.2	–	5.5	1.5	16.8	3.9	6.5	–	From *Spirulina* spp. (37% hydrol. degree)	4.0 kg ha^-1^
MAA	Mix of amino acids	45.0	5.0	1.2	50.0	5.5	0.8	24.5	9	1.5	55.0	More details in the annex	3.0 kg ha^-1^
PHE	MAA combined with pure phenylalanine	55.0	5.0	1.2	40.0	5.5	–	19.6	7.2	1.8	45.0	Phenylalanine (1%)	3.0 kg ha^-1^
ZIN	MAA combined with zinc	55.0	7.0	1.2	38.0	5.5	–	19.6	7.2	0.8	44.0	Zn (2%)	3.0 kg ha^-1^
VIT	B-group vitamins (Sigma-Aldrich, United States)	–	–	–	–	–	–	–	–	–	–	B1-thiamine (33.3%), B2-riboflavin (33.3%), B6-pyridoxine (33.3%)	1.5 kg ha^-1^
CHI	chitosan – ChitoPlant Solution (Agritalia, Italy)	98.3	0.01	–	1.7	5.2	–	–	–	–	–	–	15 kg ha^-1^
SIL	Siliforce (ILSA S.p.A., Italy)	–	–	1.2	–	2.0	–	–	–	–	–	Si (8 g kg^-1^), Zn (1.8%), Mo (0.2%)	300 ml ha^-1^


### Vegetative Growth and Leaf Gas Exchanges

Two shoots per plant were selected at a similar canopy height and position and tagged in order to measure the shoot elongation at 2-week interval, from May until growth cessation. Two fully expanded leaves from the tagged shoots were used for the indirect evaluation of the chlorophyll content with a SPAD-502 Chlorophyll Meter (Konica Minolta, Tokyo, Japan) and the measurements conducted at 2-week interval. At mid-July of both years two leaves per plant, chosen at an intermediate position along the same tagged apple shoots, were collected and leaf area was determined with a LI-COR 3000 Leaf Area Meter (LI-COR Inc., Lincoln, NE, United States). In mid-summer (28th, 30th of July and 1st of August of both years), when trees had already received nine applications of all the treatments, net assimilation (A, μmol m^-2^ s^-1^) and transpiration (E, mmol m^-2^ s^-1^) rates of leaves were evaluated using a portable gas exchange analyzer (LCpro ADC, Hoddesdon Bioscientific, Ltd., United Kingdom). The gas exchange evaluations were conducted at time 0 (T0, immediately before the tenth application of biostimulants), time 1 (T1, 48 h), and time 2 (T2, 96 h) after application. Measurements were performed on a young, fully expanded leaf of five randomly selected shoots per treatment and were taken under saturating light conditions (1,800 μmol photons m^-2^ s^-1^), around midday (11:00–13:00 h) using a broad leaf gas chamber with a window size of 6.25 cm^2^ and a flow rate of 400 ml min^-1^.

### Yield and Fruit Quality

Apples were harvested on the 1st of September of both years, approximately 140 DAFB, based on the starch-iodine maturity test. Starting from the 10th of August (approximately 20 days prior the expected time of harvest), 10 apples per replicate (40 per treatments) were randomly sampled and evaluated for their starch index (**Supplementary Figure S1**). The harvest was performed when the apples reached uniformly a value of the starch index around 2.5, which is considered the optimal picking time for the cv. Jonathan. Yield per tree was determined by collecting all fruits from three out of the five trees per replicate (those located in the central position of each replicate). Fruits from each tree were placed in tagged boxes in order to keep track of the tree they come from, and then transported in the laboratory where they were automatically sorted with a sorting machine (Aweta, Nootdorp, Netherlands). This device delivers the following parameters: number of fruits and total yield per tree, average fruit weight and red overcolor extension in percentage of apple fruit surface (four classes: <33%, 33–50%, 51–75%, and >75%). The colorimetric coordinates (L^∗^, a^∗^, and b^∗^) were determined with a colorimeter (Minolta, model CR-400, Tokyo, Japan) by measuring 10 fruits randomly selected among those belonging to the same replicate at five different positions around the equatorial side of each fruit. Values are presented as color index [CI = (1000^∗^a)/(L^∗^b)], with higher CI value indicating a more intense red color in the fruit (Tessmer et al., 2016). The total soluble solids (TSS as °Brix), titratable acidity (TA as g L^-1^ of malic acid) and flesh firmness (FF as kg cm^-2^) of 10 fruits per replicate (40 per treatment) were determined at harvest with the automatic measuring device “Pimprenelle” (Satop Giraud Technologie, Cavillon, France).

### Physiological Disorder After Storage

**FIGURE 2:**
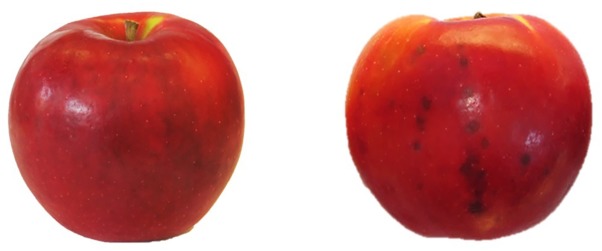
Symptoms of “Jonathan spot” disorder (**Left**: healthy fruit, **Right**: symptomatic fruit).

The tagged boxes containing around one hundred apples per tree (around 300 per replicate) were kept in cold room (2°C and RH 85–90%) and sampled regularly (every 2 months) to monitor the post-harvest ripening process. Moreover, the incidence of the physiological disorder “Jonathan spot” was evaluated during storage period by counting the number of symptomatic fruits per plant. “Jonathan spot” symptoms are characterized by irregular small black spots on the skin of apples as shown in **Figure 2**. Number of spots on each fruit (severity) can be very variable and, differently from those of the more studied apple bitter pit, the necrosis rarely involve cells of the inner pulp tissue of the apple.

### Biochemical Analysis of Apple Fruits

#### Chemicals

Ethanol (96%) was obtained from J.T. Baker (Center Valley, PA, United States). Acetic acid (96%), potassium chloride, and hydrochloric acid (36%) were from Merck (Kenilworth, NJ, United States) and Fisher Chemical (Thermo Fisher Scientific, Waltham, MA, United States), respectively. Phosphoric acid (≥99%), Folin-Ciocalteu’s phenol reagent, sodium carbonate, 2,2′-azino-bis(3-ethylbenzothiazoline-6-sulfonic acid) diammonium salt (ABTS), sodium fluoride, ascorbic acid (99%) and Trolox (6-hydroxy-2,5,7,8-tetramethylchroman-2-carboxylic acid) were purchased from Sigma-Aldrich (St. Louis, MO, United States). Sodium acetate (anhydrous) was purchased from Fisher Chemical (Thermo Fisher Scientific, Waltham, MA, United States). Potassium persulfate (K_2_S_2_O_8_) and gallic acid (≥99%) were purchased from Carl Roth (Karlsruhe, Germany). Methanol (HPLC-grade) was purchased from VWR Chemicals (Milan, Italy), and meta-phosphoric acid (≥99%) and monopotassium phosphate ( ≥ 99%) were from Thermo Fisher Scientific Inc. (Waltham, MA, United States). The ultrapure water was prepared with a Milli-Q-water purification system (EMD Millipore Corporation, Billerica, MA, United States).

#### Sample Preparation and Extraction Procedure

Six fruits per replicate (24 per treatment) were randomly collected at harvest. Apples were peeled and pulp and skin samples were sampled, immediately frozen in liquid nitrogen and stored at -80°C. Extraction was conducted using 25 mg of lyophilized sample which were homogenized and extracted in 1.8 mL of extraction solution (80% methanol acidified with H_3_PO_4_, pH 1.0) and in 30 μL of 0.1 M NaF solution for 15 min at 5°C. The extract was then filtered with PTFE filters (0.45 μm, Thermo Fisher Scientific) and the filtrate was stored at -80°C for analyses. Extraction procedure for ascorbic acid analysis is described in Section “Ascorbic Acid Quantification.”

#### Determination of Total Phenolic Content (TPC)

Total phenolic content (TPC) in peel and pulp extracts was quantified using the Folin-Ciocalteu assay following the methodology described in Wolfe et al. (2003). Briefly, a 60 μL aliquot of the sample extract was diluted with 250 μL of deionized water. Then, 60 μL Folin-Ciocalteu reagents were added and the mixture was allowed to react for 6 min at 20°C. Afterward, 630 μL of Na_2_CO_3_ (7.5% w/v) was added and incubated for 90 min at 20°C. The absorbance of samples and standards was read at 740 nm on a spectrophotometer Cary 60 UV-Vis (Agilent Technologies, Palo Alto, CA, United States) and the results were expressed as milligrams of gallic acid equivalents (GAE) per 100 grams of dry weight (mg GAE 100 g^-1^ DW) referred to a standard curve of gallic acid (range 5–500 mg L^-1^, *r*^2^ = 0.999).

#### Determination of Total Anthocyanin Content (TAC)

Total anthocyanin content (TAC) in peel extracts was determined using the spectrophotometric pH differential method as described in Lee et al. (2005). Briefly, two dilutions of the same sample were prepared by adding 200 μL of extract to 800 μL of potassium chloride (0.25 M, pH 1) and to 800 μL of sodium acetate (0.4 M, pH 4.5), respectively. The absorbances were measured at 520 and 700 nm on the Cary 60 UV-Vis spectrophotometer. Total anthocyanins content was calculated using Lambert–Beer law (𝜀 = 26900 L/mol/cm, MW = 449.2 g/mol) as mg cyanidin 3-glucoside equivalents (CGE) per 100 g of dry weight.

#### Antioxidant Activity (ABTS)

The antioxidant activity was determined using the ABTS assay as described in Re et al. (1999) with some modifications. Briefly, ABTS radical cation (ABTS^+^) was generated by reacting 7 mM of ABTS with 2.45 mM of potassium persulfate (K_2_S_2_O_8_). The mixture was incubated in a darkroom at 4°C for 16 h. ABTS^+^ solution was diluted with water until the absorbance was 0.700 ± 0.02 at 734 nm. For the assay, 30 μL of sample extract or standard were added and mixed to 1.97 mL of diluted ABTS^+^ solution. The absorbance was measured at 734 nm on the Cary 60 UV–Vis spectrophotometer after 10 min in dark conditions. A calibration curve was prepared using Trolox standard at different concentrations. The antioxidant activity results were expressed as milligrams Trolox equivalents per 100 grams of dry weight (mg Trolox 100 g^-1^ DW) using an external calibration (Trolox, range 15.6–500 mg L^-1^, *r*^2^ = 0.999).

#### Ascorbic Acid Quantification

The quantification of ascorbic acid was determined using the methodology described in Bassi et al. (2018). Briefly, an aliquot of ca. 50 mg of freeze-dried apple pulp or peel was extracted using 1 mL of extraction solution [700 μL deionized H_2_O containing 8% (v/v) acetic acid and 3% (w/v) metaphosphoric acid added with 300 μL of methanol] (AOAC, 2005), mixed using a Vortex-Genie 2 at 3200 rpm for about 20 s at room temperature and filtered through a 0.20 μm PTFE filter. An HPLC Agilent 1260 Infinity (Santa Clara, CA, United States) with a diode array (1260 DAD VL) detector was used for the analysis of the ascorbic acid. Chromatographic separation was carried out at 25°C using a Kinetex 5 μ C18 100 Å column (150 mm × 4.6 mm, 5 μm particle size; Phenomenex, Torrance, CA, United States) and a pre-column (4.6 mm, Security Card, Phenomenex, Torrance, CA, United States). The detection wavelength was fixed at 260 nm. The mobile phases used were 5 mM KH_2_PO_4_, pH 4.8 (solvent A) and methanol (solvent B). The analytical gradient was the follow: 0 min, 100% A; 2.5 min, 100% A; 6 min, 80% A; 8 min, 100% A, and 13 min, 100% A. The flow rate was set at 1.0 mL min^-1^. The temperature of the autosampler was 4°C and injection volume was 5 μL. The Agilent ChemStation^TM^ (ver. C.01.03) (Agilent Technologies, Palo Alto, CA, United States) was used for system control and data processing.

#### Mineral Elements in Fruit Skin

At harvest of both years, six fruits per replicate (four replicates per treatment) were randomly selected and peeled. Skin samples were immediately frozen in liquid nitrogen and stored at -80°C. Subsequently, samples were lyophilized, ground and homogenized for mineral element content analyses. Nitrogen (N) content was determined by Kjeldahl method and the other macro (P, K, Ca, and Mg) and microelements (S, Fe, Cu, B, Zn, Mn, Na, and Si) were analyzed by using the inductively coupled plasma optical emission spectrometry (ICP-OES).

### Statistical Analysis

A two-way analysis of variance (ANOVA) on the complete randomized block design was performed on the data, using the factors “treatment” and “year” as fixed and the factor “block” as random. Mean separation of variables with equal variance was performed by the Dunnett’s test, contrasting each biostimulant group mean against the control group mean. This procedure is recommended when working with several experimental treatments (Dunnett, 1985). Data expressed in percentage (classes of red overcolor extension and “Jonathan spot” incidence) were arcsine-transformed prior to the application of the ANOVA. In case of significant interaction between the factor “treatment” and the factor “year,” results were presented separately for the 2 years in dedicated figures as vertical grouped bars with standard error per treatment combination. Data gathered from repeated measurements during both seasons (shoot length, SPAD index, leaf gas exchanges, and incidence of post-harvest disorder) were subjected to one-way ANOVA for single year, using the Dunnett’s test to compare the means at each data point.

## Results

### Vegetative Growth

**FIGURE 3:**
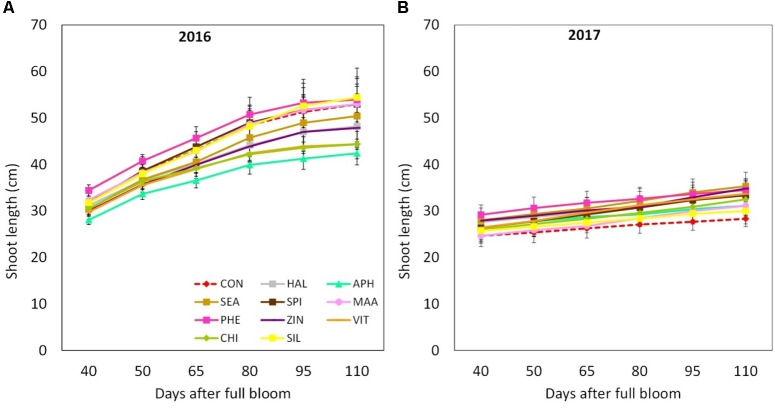
Shoot growth dynamics (from 40 to 110 DAFB) in apple plants treated with different biostimulant products and water (control) for year 2016 **(A)** and 2017 **(B)**. Vertical bars indicate mean ± SE, *n* = 4. Treatments’ legend: CON, control; HAL, humic acids; APH, alfalfa protein hydrolysate; SEA, macroseaweed extract; SPI, microalga hydrolysate; MAA, mix of amino acids; PHE, MAA combined with pure phenylalanine; ZIN, MAA combined with zinc; VIT, B-group vitamins; CHI, chitosan; SIL, Siliforce^®^.

**FIGURE 4:**
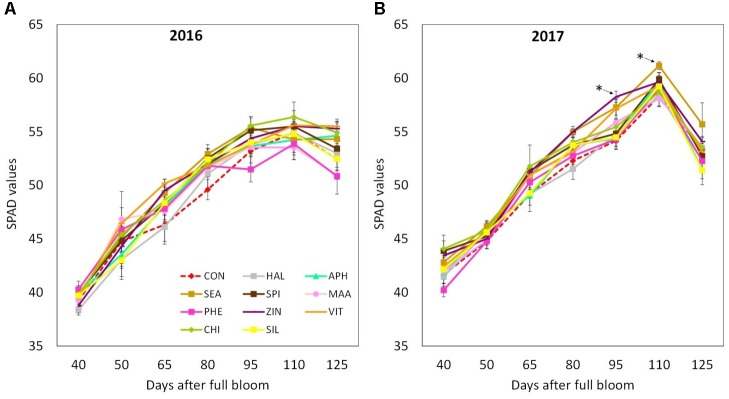
Chlorophyll content (as SPAD values) dynamics (from 40 to 125 DAFB) in apple plants treated with different biostimulant products and water (control) for year 2016 **(A)** and 2017 **(B)**. Vertical bars indicate mean ± SE, *n* = 4. ^∗^Indicates significant differences according to Dunnett’s test. ^∗∗∗^*P* < 0.001; ^∗∗^*P* < 0.01; ^∗^*P* < 0.05.

Final shoot growth resulted significantly affected by the factor “year,” 2016 being generally characterized by longer shoots (40–50 cm) at the end of the growing season as compared to year 2017 (25–35 cm) (**Figures 3A,B**). Treatments with biostimulants did not induce any significant modification of the growth dynamic in both years (**Figure 3**). Leaf area was generally larger in year 2017 than in 2016 (**Table 2**). Independently from the considered year, SEA applications were found able to increase the average leaf area by around 20%. Chlorophyll content (measured as SPAD index) reached slightly higher values in year 2017 than in year 2016 (**Figures 4A,B**). Treatments did not induced any significant modification of the chlorophyll content in year 2016, whereas leaves treated with ZIN and SEA showed higher SPAD values than control in year 2017 at 95 and 110 DAFB, respectively (**Figure 4B**).

**Table 2:** Leaf area and fruit yield parameters at harvest as affected by biostimulants and growth season.

	Leaf area (cm^2^)	Yield (kg tree^-1^)	Number fruits tree^-1^ (N.)	Fruit weight (g)	Fruit diameter (mm)
**Treatment**					
CON	26.30 ± 0.78^1^	14.61 ± 1.62	96.67 ± 13.54	157.45 ± 6.27	72.51 ± 0.87
HAL	25.92 ± 0.79	15.44 ± 0.93	97.50 ± 6.88	161.21 ± 4.00	73.00 ± 0.59
APH	28.80 ± 1.92	13.79 ± 0.78	86.92 ± 4.93	161.35 ± 4.86	73.10 ± 0.71
SEA	32.22 ± 0.97^∗∗^	12.75 ± 0.82	78.21 ± 5.78	165.20 ± 4.35	73.73 ± 0.64
SPI	30.25 ± 0.99	13.99 ± 1.02	88.67 ± 7.67	160.11 ± 3.80	73.00 ± 0.54
MAA	28.16 ± 0.84	13.50 ± 0.88	90.33 ± 8.25	158.48 ± 6.88	72.81 ± 1.05
PHE	26.91 ± 1.05	15.43 ± 1.33	95.67 ± 9.65	165.53 ± 3.99	73.91 ± 0.60
ZIN	30.24 ± 1.30	13.08 ± 0.79	81.42 ± 5.59	163.79 ± 5.42	73.62 ± 0.80
VIT	29.82 ± 1.35	12.76 ± 0.96	76.33 ± 5.98	169.99 ± 4.05	74.68 ± 0.54
CHI	27.20 ± 0.82	13.79 ± 1.36	88.54 ± 10.30	160.03 ± 4.41	73.01 ± 0.71
SIL	27.83 ± 1.34	15.52 ± 1.11	100.21 ± 10.45	159.05 ± 5.42	72.70 ± 0.81
**Significance**	^∗∗^	ns	ns	ns	ns
**Year**					
2016	27.79 ± 1.34	14.38 ± 0.90	84.64 ± 5.59	171.67 ± 2.44	74.59 ± 0.38
2017	29.24 ± 1.16	13.74 ± 1.24	93.62 ± 10.35	152.36 ± 4.18	71.97 ± 0.68
**Significance**	^∗^	ns	^∗∗∗^	^∗∗∗^	^∗^
T × Y	ns	ns	ns	ns	ns


^1^Mean ± SE. Values followed by asterisk indicate significant differences between a single treatment group and control group, according to Dunnett’s test (n = 4). ***P < 0.001; **P < 0.01; *P < 0.05, ns, not significant.

### Leaf Gas Exchanges

**FIGURE 5:**
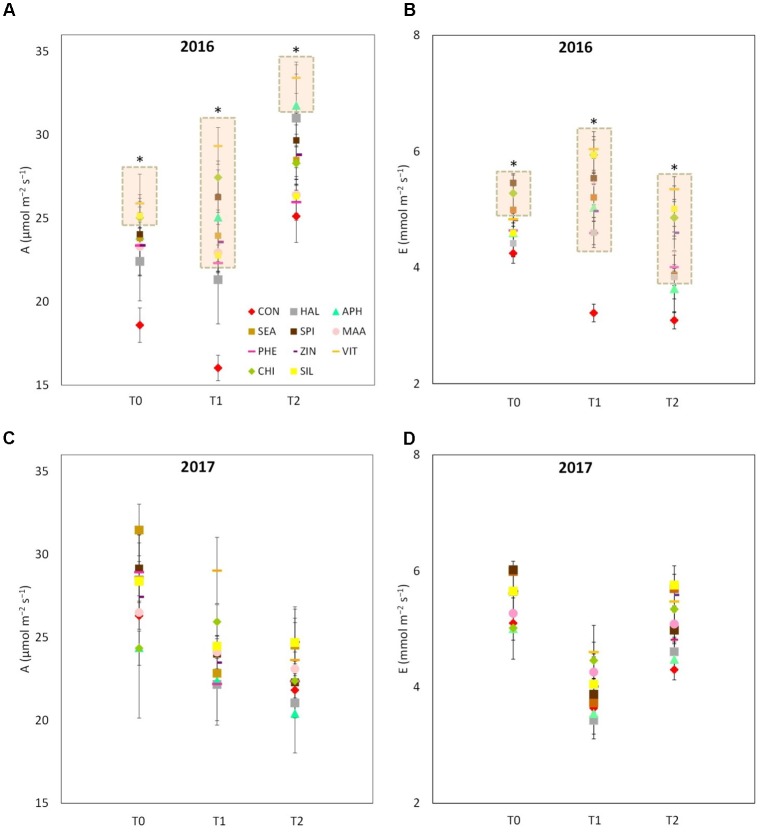
Photosynthetic and transpiration rates measured in 2016 (**A,B**, respectively) and 2017 (**C,D**, respectively) as affected by biostimulant applications (T0: immediately before spray; T1: 48 h after spray; T2: 96 h after spray). Asterisk (^∗^) and the pink rectangle in the background indicate the group of treatments that significantly differed from control according to Dunnett’s test (*P* < 0.05).

VIT, SIL and CHI showed higher leaf photosynthetic rates as compared with control at T0 of year 2016 (**Figure 5A**). Forty-eight hours after the sprays (T1), leaves from all treatments (with the exception of HAL) presented a higher photosynthetic rate (values ranging from 20 to 27 μmol m^-2^ s^-1^) as compared to control (15 μmol m^-2^ s^-1^). Four day after the application of the biostimulants (T2), the differences in the leaf photosynthetic rates among treatments were reduced, even though APH and VIT were still significantly higher than control. Similarly to the photosynthetic rate, leaf transpiration of treated trees resulted significantly higher than control in 2016, especially 48 and 96 h after the sprays (**Figure 5B**). In 2017, photosynthetic rate and transpiration presented values within the same range as in 2016, but no significant differences were detected between control leaves and leaves previously treated with the biostimulants (**Figures 5C,D**).

### Yield and Fruit Quality

Fruits per tree were less numerous in 2016 than in 2017, but of significantly higher weight (170 vs. 150 grams per fruit approximately, **Table 2**). Overall, the final yield per tree did not differ between years, the average value being around 14 kg tree^-1^. As for the effect of biostimulants on yield and fruit biometric characteristics (weight and diameter), no statistically significant differences were shown (**Table 2**).

**FIGURE 6:**
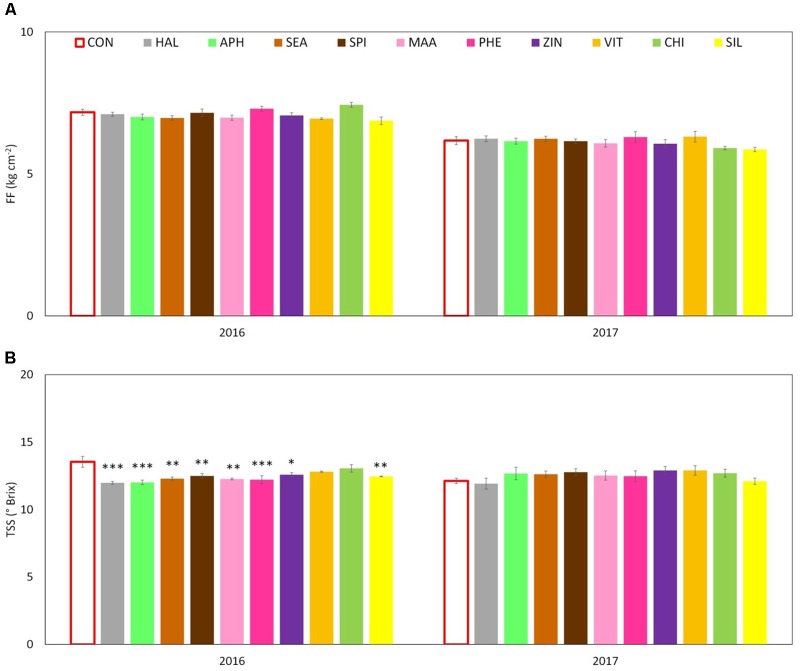
Effect of the biostimulants on flesh firmness – FF **(A)** and total soluble solids – TSS **(B)** compared with control for year 2016 and 2017. Vertical bars indicate mean ± SE, *n* = 4. ^∗^Indicates significant differences according to Dunnett’s test. ^∗∗∗^*P* < 0.001; ^∗∗^*P* < 0.01; ^∗^*P* < 0.05.

**FIGURE 7:**
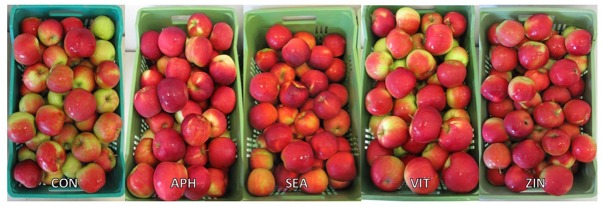
Apple color at harvest as affected by treatment applications (CON, control; APH, alfalfa protein hydrolysate; SEA, macroseaweed extract; VIT, B-group vitamins; ZIN, zinc plus amino acids).

**FIGURE 8:**
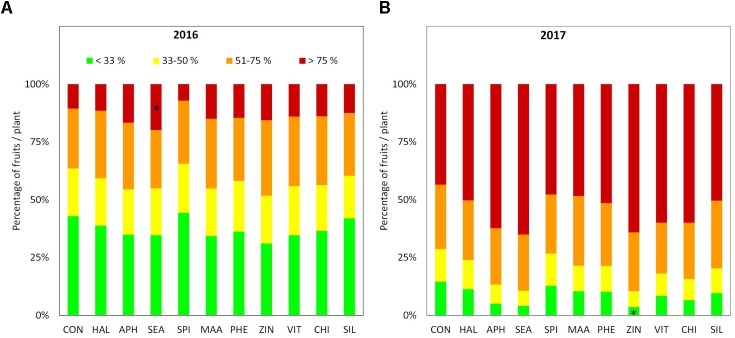
Percentage of fruits according to red overcolor extension classes at harvest 2016 **(A)** and 2017 **(B)**, *n* = 4. ^∗^Indicates significant differences according to Dunnett’s test. ^∗∗∗^*P* < 0.001; ^∗∗^*P* < 0.01; ^∗^*P* < 0.05.

Apples were harvested at the same ripening stage in year 2016 and 2017 as shown by very similar values of the average starch index for both seasons (**Supplementary Figure S1**). As for FF and TA, their average values were found higher in 2016 than in 2017, whereas biostimulant applications were ineffective on these parameters (**Table 3** and **Figure 6A**). TSS at harvest presented values ranging from 12 and 13.5°Brix. Apples from most of the biostimulant-treated trees (HAL, APH, SEA, SPI, MAA, PHE, ZIN, and SIL) were characterized by values of TSS approximately 1–1.5 degree lower than control in year 2016 only (no differences in 2017) (**Figure 6B**). Treatments with selected biostimulants had a visible and significant effect on the final fruit color index, independently from the considered year (**Figure 7**). More in detail, apples previously treated with the SEA, APH, and, to a less extent, by VIT, were characterized by a most intense red over coloration and presented values of the color index that were significantly higher than control (**Table 3**). Red overcolor was significantly more pronounced in 2017 than in 2016 with more than 50% of the apples belonging to the most colored class (>75%) (**Figures 8A,B**). Among the tested biostimulants, SEA was significantly effective in enhancing the percentage of fruits presenting more than 75% overcoloration (+87% and +50% as compared to control in 2016 and 2017, respectively). As for the other treatments, APH and ZIN slightly improved red overcolor of fruits, even though not significantly.

**Table 3:** Fruit quality traits (TA, titratable acidity; CI, color index; TAC, total anthocyanin content; AA, ascorbic acid in the pulp) as affected by biostimulants and growth season.

	TA (g L^-1^)	CI	TAC (mg CGE 100 g^-1^ DW)	AA (mg 100 g^-1^ DW)
**Treatment**				
CON	6.16 ± 0.231	12.54 ± 1.51	58.07 ± 8.37	19.56 ± 3.86
HAL	6.86 ± 0.20	13.91 ± 1.95	75.05 ± 12.79	20.49 ± 4.33
APH	6.54 ± 0.29	31.15 ± 2.17***	125.50 ± 12.89***	15.76 ± 2.62
SEA	6.93 ± 0.32	31.44 ± 2.27***	186.11 ± 9.29***	16.25 ± 2.28
SPI	7.30 ± 0.12	16.49 ± 2.32	126.26 ± 12.02***	16.33 ± 2.94
MAA	6.74 ± 0.35	13.61 ± 2.02	74.73 ± 6.90	20.43 ± 3.70
PHE	6.98 ± 0.17	21.35 ± 3.40	106.84 ± 15.59**	19.09 ± 3.25
ZIN	6.81 ± 0.27	20.99 ± 2.37	113.76 ± 19.16**	20.12 ± 4.26
VIT	7.14 ± 0.38	22.67 ± 3.76*	137.92 ± 10.57***	14.07 ± 2.21
CHI	6.18 ± 0.90	21.31 ± 2.21	124.68 ± 14.37***	15.94 ± 2.71
SIL	6.65 ± 0.31	15.84 ± 2.42	78.59 ± 8.57	21.76 ± 4.90
**Significance**	*ns*	***	***	*ns*
**Year**				
2016	7.26 ± 0.15	20.38 ± 2.65	126.31 ± 13.33	25.53 ± 2.98
2017	6.24 ± 0.44	19.85 ± 3.73	93.24 ± 18.42	10.80 ± 0.66
**Significance**	***	*ns*	***	***
				
T × Y	*ns*	*ns*	*ns*	*ns*


^1^Mean ± SE. Values followed by asterisk indicate significant differences between a single treatment group and control group, according to Dunnett’s test (n = 4). ***P < 0.001; **P < 0.01; *P < 0.05, ns, not significant.

**FIGURE 9:**
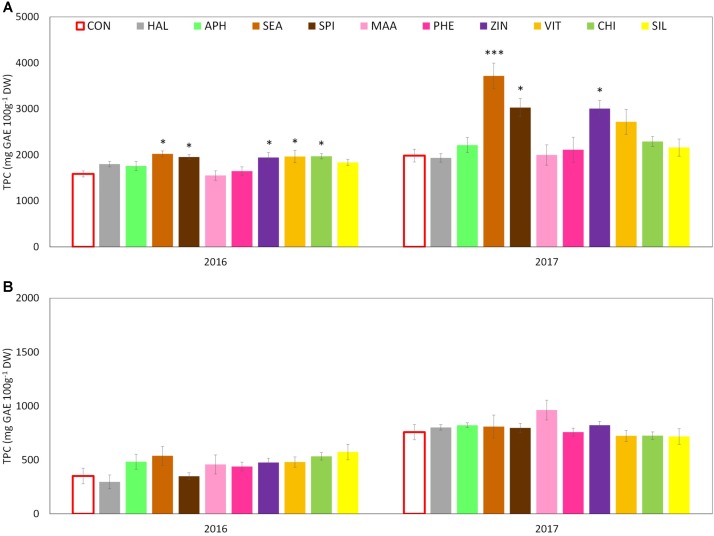
Effect of the biostimulants on total phenolic content (TPC) in apple skin **(A)** and pulp **(B)** compared with control for year 2016 and 2017. Vertical bars indicate mean ± SE, *n* = 4. ^∗^Indicates significant differences according to Dunnett’s test. ^∗∗∗^*P* < 0.001; ^∗∗^*P* < 0.01; ^∗^*P* < 0.05

**FIGURE 10:**
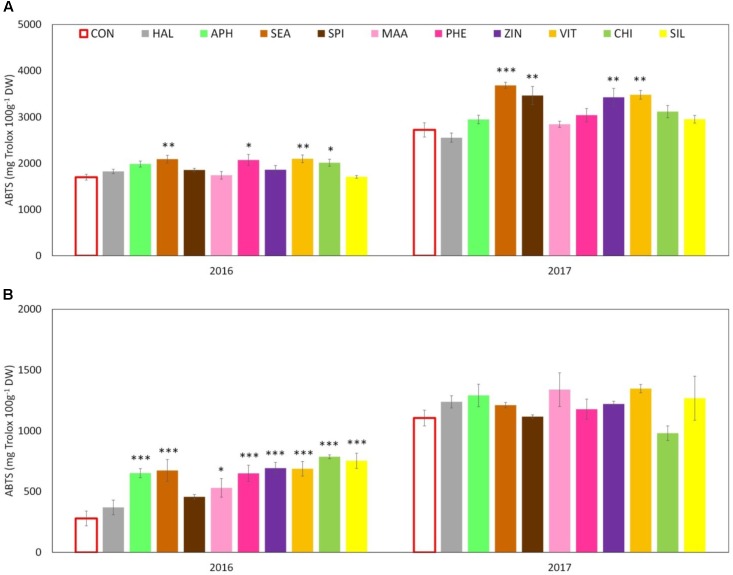
Effect of the biostimulants on antioxidant activity (as ABTS) in apple skin **(A)** and pulp **(B)** compared with control for year 2016 and 2017. Vertical bars indicate mean ± SE, *n* = 4. ^∗^Indicates significant differences according to Dunnett’s test. ^∗∗∗^*P* < 0.001; ^∗∗^*P* < 0.01; ^∗^*P* < 0.05.

**FIGURE 11:**
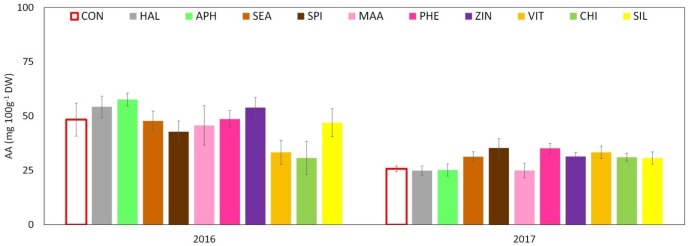
Effect of the biostimulants on ascorbic acid (AA) level in apple skin compared with control for year 2016 and 2017. Vertical bars indicate mean ± SE, *n* = 4. ^∗^Indicates significant differences according to Dunnett’s test. ^∗∗∗^*P* < 0.001; ^∗∗^*P* < 0.01; ^∗^*P* < 0.05.

Total phenolic content (TPC) evaluated at skin level was significantly affected by both factors (“treatment” and “year”) and by their interaction. The total amount was significantly higher in 2017 than in 2016 with average concentrations of around 2,400 and 1,800 mg GAE 100 g^-1^ DW, respectively. Treatments with SEA, SPI, and ZIN significantly enhanced TPC in apple skin at harvest in both years, whereas CHI and VIT were effective in 2016 only (**Figure 9A**). TPC at apple pulp level was also higher in 2017 than in 2016, without any significant effect of the treatments in both years (**Figure 9B**). TAC of apple skin was higher in year 2016 as compared to year 2017 (**Table 3**). TAC was also significantly enhanced by selected biostimulants, independently from the considered year (no significant interaction “T × Y”). More in detail, in apples treated with APH, SEA, SPI, VIT, and CHI the final anthocyanin concentration was more than the double of that of control. The antioxidant potential (ABTS) of apple skin and pulp tissues was linked to the phenolic content of these tissues. Similarly to the TPC parameter, ABTS was significantly higher in 2017 with values that were almost double than those of the year 2016 in both skin and pulp (**Figures 10A,B**). Treatments with SEA and VIT were able to significantly increase ABTS at skin level in both years, whereas other biostimulants (PHE, CHI, SPI, and ZIN) enhanced the antioxidant potential of apple skin in only one of the two considered years (**Figure 10A**). Similarly to the skin tissue, pulp antioxidant potential was significantly affected by the factors “treatment,” “year” and their interaction. Pulp antioxidant potential was enhanced by treatments in 2016 only (**Figure 10B**), with CHI and SIL among the most effective biostimulants in promoting this feature. Treatments did not affect the final ascorbic acid concentration in both apple skin and pulp (**Figure 11** and **Table 3** for skin and pulp, respectively). The factor “year” was the only significantly relevant for final ascorbic acid accumulation, year 2016 showing approximately a twofold concentration in both skin and pulp tissues as compared to 2017.

### Incidence of Physiological Disorder After Cold Storage

**FIGURE 12:**
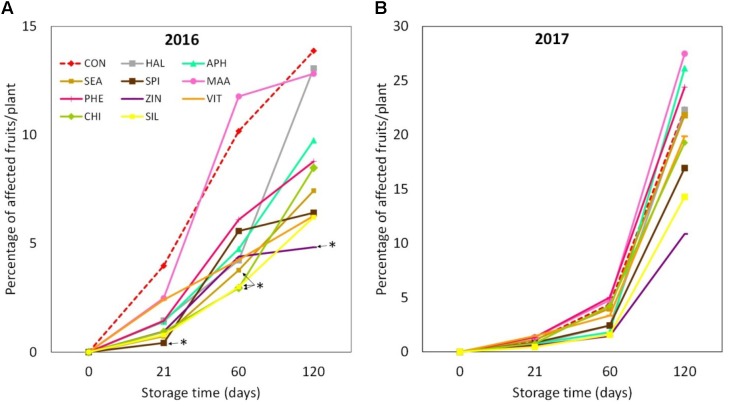
“Jonathan spot” incidence during apple storage in 2016 **(A)** and 2017 **(B)**, *n* = 4. ^∗^Indicates significant differences according to Dunnett’s test. ^∗∗∗^*P* < 0.001; ^∗∗^*P* < 0.01; ^∗^*P* < 0.05.

“Jonathan spot” incidence was significantly higher in 2017 apples, when around 20% of the fruits collected from each considered tree were symptomatic after 4 months (**Figures 12A,B**). In 2016, selected biostimulants (SPI, CHI, SIL, and SEA) were able to significantly reduce the incidence of the “Jonathan spot” disorder during different phases of the post-harvest of apples (+21 and +60 days from harvest). At the end of the storage period (4 months after harvest) apples treated with ZIN were the only ones showing a significantly lower incidence of the disorder, with a reduction of approximately 60% as compare to control (**Figure 12A**). In 2017 biostimulant applications were not as effective as in 2016. ZIN, SIL, and SPI slightly reduced the disorder incidence at the end of the 4 months storage even though differences with control were not statistically significant (**Figure 12B**).

## Discussion

The vegetative growth behavior of apple trees was mainly determined by the seasonal climatic conditions. Despite trees were drip irrigated during both seasons, warmer meteorological conditions during May–July 2017 (high maximal and average temperatures, limited rainfalls in May, **Figure 1**) might have determined a stressful status for trees, which then reduced shoot growth (**Figure 3**). In such conditions, trees treated with SEA were characterized by similar shoot growth but larger leaf area as compared to untreated trees (**Figure 3** and **Table 2**). Seaweed extracts were found able to promote shoot length in apple cv. Fuji (Spinelli et al., 2009) and to contrast drought stress effects on vegetable crops such as tomato and spinach by enhancing their growth and foliar density (Xu and Leskovar, 2015; Goñi et al., 2018). Another effect of SEA application was the higher concentration of chlorophyll (measured with the SPAD index) shown by treated leaves as compared to the untreated ones in 2017 (**Figure 4B**). This result is in agreement with the outcomes of other experiments conducted with seaweeds on apple (Spinelli et al., 2009) and on other crops such as grapevine (Sabir et al., 2014), cabbage (Rengasamy et al., 2016), and tomato (Goñi et al., 2018). This represents therefore a further evidence of a possible role of seaweed extracts in the reduction of chlorophyll degradation and in delaying leaf senescence (Battacharyya et al., 2015). As for the gas exchanges at leaf level, the first measurement was conducted when trees had already received nine biostimulant applications (every week starting from 40 DAFB). This might explain the higher A and E rates shown by the biostimulant-treated leaves before (T0) and after (T1 and T2) the 10th spray in year 2016 (**Figures 5A,B**). Photosynthetic and transpiration rates did not differ between biostimulants and control in 2017 (**Figures 5C,D**). Moreover, despite the higher values of chlorophyll concentration detected in SEA-treated leaves at the end of the 2017 season, their photosynthetic activity was not enhanced on that year. These results are partially different from those reported by Spinelli et al. (2009) on “Fuji” apple trees, where a consistent increase of chlorophyll content and photosynthetic activity was detected after the application of a commercial seaweed extract. Beside the different conditions that could have characterized the plants at the time of the measurements (leaf temperature, leaf water status, stomatal conductance, etc.), also the different methodology used for the analysis of the gas exchanges (punctual measurements at leaf level vs. continuous measurements at whole canopy level) might explain this partial inconsistency. In addition, according to values reported in the literature (Jakopic et al., 2007), the chlorophyll content was above the sufficiency threshold for apple leaves, leaving the photosynthetic rate more dependent from other environmental or endogenous factors. The only biostimulant showing a rather consistent effect on photosynthetic rate was the B-group vitamins, which was able to increase the photosynthetic potential of treated leaves in both years (**Figures 5A,C**). The use of single vitamin B1 (thiamine) was found ineffective on photosynthetic rate of rice leaves (Bahuguna et al., 2012); comparison with the outcome of the present research is anyway difficult giving that a complex mix of vitamins (including B1, B2, and B6) and not the single thiamine was used in our study.

At harvest, no difference was found on tree productivity in both years (**Table 2**). This outcome partially differs from another research performed on apple (Spinelli et al., 2009) where the use of a similar seaweed extract (from *Ascophyllum nodosum*) was found able to induce a higher final yield in a year of low crop load. The protein hydrolysate from alfalfa resulted also ineffective on final yield (**Table 2**). Differently, other studies conducted on vegetable crops (Rouphael et al., 2017 and Polo and Mata, 2018 on tomato; Ertani et al., 2015 on hot pepper) showed an increment of plant productivity, probably as the result of a stimulation mechanism of the plant primary metabolism triggered by signaling molecules (peptides, oligopeptides, and free amino acids) contained in the hydrolysate.

The effect of the biostimulants on primary apple quality traits (FF, TSS, and TA) was limited (**Figure 6** and **Table 3**). Only in 2016, the fruit TSS was found lower in treated apples (**Figure 6B**). According to the available literature, biostimulants can have different effect on final sugar accumulation in fruits. Protein hydrolysate-based substances were found able to enhance final sugar content in hot pepper and tomato (Ertani et al., 2015; Rouphael et al., 2017). Differently, seaweed extracts did not changed or slightly reduced final Brix value in strawberry fruits (Roussos et al., 2009) and grapevine berries (Frioni et al., 2018). In our conditions, the lower TSS could be the result of an internal trade-off at fruit level for carbon skeleton between sugars and secondary metabolites (i.e., phenolic compounds) that might had occurred in year 2016, as also suggested by the works of Lux-Endrich et al. (2000) and Rühmann et al. (2002) on apple.

One of the major effect of treatments application was the significant change in final apple coloration obtained with SEA, APH, and VIT. This outcome was confirmed by the colorimetric coordinates (color index), by the total anthocyanin concentration measured on fruit samples taken from different replications and by the evaluation of the red overcolor extension performed on all fruits harvested from all the considered trees under evaluation (**Table 3** and **Figures 7**, **8**). These results confirm those described by Malaguti et al. (2002) on apple “Gala” and those by Frioni et al. (2018) on red grapevine cultivars evaluated in different cultivation areas. The boosted final red over color of apples might be ascribed to a modulation of the metabolism of plant endogenous growth regulators (mainly cytochins and abscisic acid) obtained with the application of the biostimulant substances (i.e., *A. nodosum*; Wally et al., 2013), leading to an induction of anthocyanin biosynthesis and accumulation in fruit skin prior to harvest (**Table 3**).

Another relevant effect of the biostimulant applications was the higher concentration of phenolic compounds detected in the skin tissue of apples treated with SEA, SPI, and ZIN in the 2 years (**Figure 9A**). Since no difference was found for the ascorbic acid concentration, this increase in phenolic compounds was likely responsible for the higher ABTS values showed by skin samples of fruits previously treated with the biostimulants mentioned above (**Figure 10A**). Phenolic compounds are biologically active metabolites showing antioxidant potential (Rice-Evans et al., 1997) and therefore highly considered as health-promoting substances in fruits (Slavin and Lloyd, 2012). Similar health-promoting responses were found in onion and potato after the application of *A. nodosum* (Lola-Luz et al., 2014). Protein hydrolysates of different origin (legume and alfalfa) promoted the antioxidant capacity of tomato (Rouphael et al., 2017) and green or red pepper (Ertani et al., 2015) similarly to what was detected for the apple pulp in our study (**Figure 10B**). It has been shown that the protein hydrolysate mode of action involves the up-regulation of a number of genes responsible for the secondary metabolism of plants leading to the synthesis and accumulation of phenolics and terpenes which are responsible for the enhanced antioxidant activity and for the increased tolerance to biotic and abiotic stresses (Ertani et al., 2017). Moreover apple treated with APH were also characterized by a higher total anthocyanin and a lower nitrogen content at skin level (**Table 3** and **Supplementary Table S2**). A negative correlation between the concentration of nitrogen and the concentration of anthocyanin and total flavonoids was often found when measured in apple skin tissue (Awad and de Jager, 2002). Low nutrients concentration at skin level are often positive for the final coloration of fruits, probably as a result of the internal trade-off between the synthesis of secondary substances and the growth primary metabolism as also described by Veberic (2016). Higher phenolic concentration and antioxidant activity were also detected in apple skin after B-group vitamins application (**Figures 9A**, **10A**). Similar results were obtained with vitamin B1 (thiamine) on grapevine. It was found that thiamine was able to elicit different genes belonging to the phenylpropanoid pathway (including phenylalanine ammonia lyase, chalcone synthase, and stilbene synthase) leading to a higher accumulation of secondary metabolites and antioxidant activity with positive effect on tolerance to downy mildew in grapevine (Boubakri et al., 2013). Zinc applications also induced phenolic compounds accumulation and antioxidant potential in apple (**Figures 9A**, **10A,B**). This result partially conflicts with those of Aglar et al. (2016) which described a negative effect of the application of zinc on both phenols and antioxidant potential in apple. Studies on the role of zinc on the apple phenolic metabolism are currently not available in the literature. An interpretation of this conflicting result could consider the different product formulation used in the present study (a mix of zinc and amino acids), whereas it was zinc sulfate alone in the case of Aglar et al. (2016). Moreover, differences in the cultivars studied and in the experimental conditions could also have played a role on the outcomes of the experiments. Anyhow, the role of genotype in plant response to zinc treatments seems to be of relevance, considering that the phenolic accumulation after zinc sulfate application was found enhanced in grapevine (Song et al., 2015) and in aromatic herbs extracts such as dill and anise, but reduced in other *species* such as fennel (Majdoub et al., 2017).

Nutrient concentration at the skin tissue level has often been linked to the incidence of physiological disorders in apple (Amarante et al., 2013; Baugher et al., 2017). In our experiment, the application of zinc (in combination with a mix of amino acids) was found effective in lowering the incidence of the physiological disorder of apple during storage (**Figure 12**). This effect was consistent in both years, even though incidence percentage was significantly higher in 2017 probably because of the higher rainfalls that characterized the month of July and August of that year. Deficit in nutrient concentration in fruit skin (calcium in particular) as well as adverse meteorological conditions are commonly associated with post-harvest disorders (Ferguson et al., 1999). The role of other mineral nutrients (such as zinc and silicon) for fruit storability is anyway still unclear. Pais and Petho (1970) found that foliar applications of calcium plus zinc retarded the development of post-harvest disorder in apple cv. Jonathan. Zinc applications at 10-day intervals from 8 weeks after full bloom to harvest were found to reduce loss of mechanical properties of apples after storage (Johnson and Dover, 2002). The authors explained this result as a possible indirect effect of zinc applications on the enhanced calcium concentration of fruits, which likely had a positive consequence on apple storability. In our study, apples treated with zinc in the form of zinc + amino acids were found significantly richer in zinc and showed a tendency to higher calcium concentration (**Supplementary Tables S2**, **S3**). Both these elements may have contributed to strengthen the middle lamella and primary cell wall structure (Henriques et al., 2012; Guerriero et al., 2016), therefore contrasting the development of the post-harvest disorder “Jonathan spot” during storage period.

## Conclusion

This study is the first comprehensive investigation of the use of different classes of biostimulants on organic apple production over a period of two consecutive years. The results of the study indicate that products based on alfalfa protein hydrolysate, seaweed extracts and B-group vitamins improved final red coloration of apple “Jonathan” in both years, therefore enhancing their market potential. Moreover, the same biostimulants had a positive effect on fruit functional traits as shown by the higher phenolic compounds concentration, total anthocyanin and antioxidant potential of treated apples. Taken together, these findings suggest a role for selected biostimulants in promoting the secondary metabolism of treated plants, leading to an improvement of fruit quality, appearance, and nutritional value. The research has also shown that biostimulants containing zinc are effective in reducing the incidence of physiological disorders in cold stored apples, strengthening the idea of a positive role of this element on the structure and resistance of cell walls and membranes at fruit level. Giving the current lack of effective means for the post-harvest management of organic fruits, this finding might have significant implication on the practices presently used for organic apple conservation and commercialization. As for the fine tuning of the use of biostimulants under orchard conditions, further research needs to be done to deepen our understanding on the way of application of the available products (number of applications, period, and concentrations) for the different apple cultivars on the market.

## Author Contributions

SS contributed in the set-up of the experimental protocol, performed the agronomic study, processed the fruit samples, analyzed the data, and participated to the writing of the paper. MK contributed to the set-up of the experiment and to the interpretation of the results. CC contributed to the agronomic implementation of the study. MB contributed to the analysis of phenols, anthocyanins, antioxidant potential, and ascorbic acid. PR contributed to the set-up of the analytical work related to the quality indexes of the fruits. CA coordinated the research, worked on the statistical analysis, and contributed to the writing of several sections of the manuscript.

## Conflict of Interest Statement

ILSA S.p.A. provided part of the biostimulants used for the experiment. Collection of data, analysis and interpretation, as well as article writing, was carried out by the authors independently. The authors declare that the research was conducted in the absence of any commercial or financial relationships that could be construed as a potential conflict of interest. The authors declare that the research was conducted in the absence of any commercial or financial relationships that could be construed as a potential conflict of interest.
