# Supplementary material for: Use of Biostimulants for Organic Apple Production: Effects on Tree Growth, Yield, and Fruit Quality at Harvest and During Storage
Source: Front Plant Sci. 2018 Sep 20;9:1342. doi: 10.3389/fpls.2018.01342 (PMC6160664; doi:10.3389/fpls.2018.01342)
Supplement: Supplementary file 1 [file Data_Sheet_1.docx]

ANNEX

FIGURES


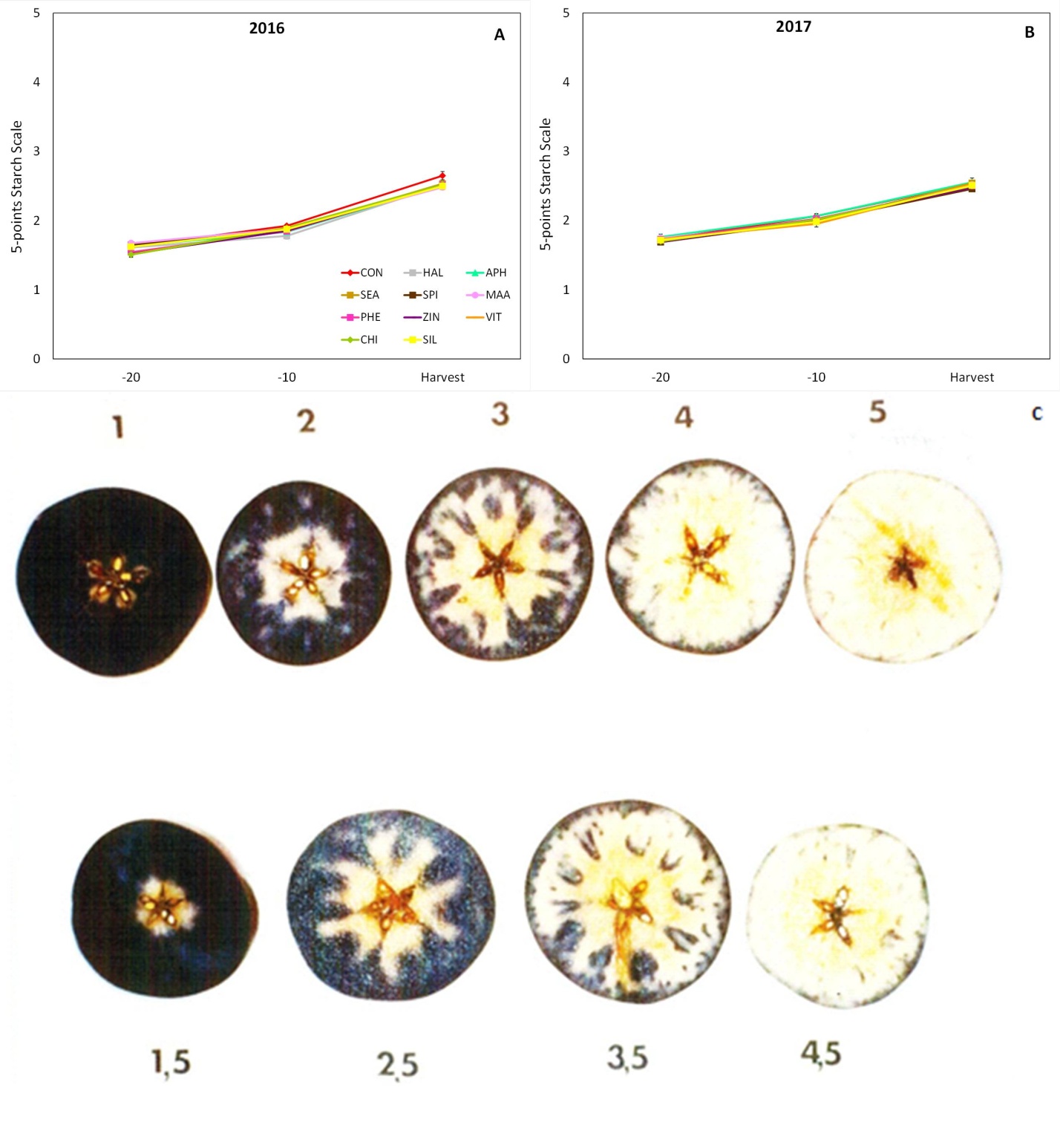
 Figure S1. Starch degradation dynamic for years 2016 (A) and 2017 (B). Starch degradation was assessed according to a 5-points scale (C) specifically adapted to the cv Jonathan. Values around 2.5 are considered optimal for the commercial harvest of the cv. Jonathan.

TABLES

Table S1. Additional information on amino acids composition of alfalfa protein hydrolysate (APH) and the mix of amino acids (MAA)

| Total amino acids (% w w^-1^) | APH | MAA |
| --- | --- | --- |
| Aspartic Acid | 0.99 | 3.20 |
| Glutamic Acid | 0.74 | 5.64 |
| Alanine | 0.41 | 4.95 |
| Arginine | 0.06 | 4.32 |
| Phenylalanine | 0.16 | 1.11 |
| Glycine | 0.36 | 12.80 |
| Hydroxyproline | 0.11 | 6.11 |
| Isoleucine | 0.21 | 0.82 |
| Histidine + Glutamine | 0.05 | 0.42 |
| Leucine | 0.30 | 1.64 |
| Lysine | 0.13 | 1.98 |
| Proline | 0.63 | 7.45 |
| Serine + Asparagine | 0.18 | 1.89 |
| Tyrosine | 0.08 | 0.33 |
| Threonine | 0.18 | 0.82 |
| Valine | 0.33 | 1.22 |
| Metionine | 0.05 | 0.44 |
| Cysteine | 0.08 | <0.01 |
| Tryptofane | 0.06 | <0.01 |
|  |  |  |
| Total | 5.11 | 55.1 |

Table S2. Macro-nutrients content of apple skin as affected by treatments with biostimulants and growth season

|  | N (% DW) | | P (mg/100 g DW) | | K (mg/100 g DW) | | Ca (mg/100 g DW) | | Mg (mg/100 g DW) | | S (mg/kg DW) | |
| --- | --- | --- | --- | --- | --- | --- | --- | --- | --- | --- | --- | --- |
| Treatment |  |  |  |  |  |  |  |  |  |  |  |  |
| CON | 0.47 | ± 0.03^1^ | 61.35 | ± 3.41 | 833.57 | ± 27.74 | 45.63 | ± 2.37 | 92.97 | ± 2.95 | 508.19 | ± 32.27 |
| HAL | 0.43 | ± 0.02 | 59.15 | ± 2.23 | 796.10 | ± 25.52 | 47.01 | ± 2.51 | 91.62 | ± 3.23 | 475.97 | ± 16.75 |
| APH | 0.38 | ± 0.03 *** | 53.89 | ± 2.75 | 710.10 | ± 8.77 ** | 39.10 | ± 1.83 | 84.12 | ± 4.02 | 406.20 | ± 17.56 *** |
| SEA | 0.42 | ± 0.02 | 53.20 | ± 2.01 | 726.79 | ± 20.01 * | 43.80 | ± 4.29 | 91.99 | ± 2.30 | 455.84 | ± 16.70 |
| SPI | 0.47 | ± 0.02 | 57.46 | ± 0.97 | 786.60 | ± 30.94 | 49.59 | ± 4.12 | 96.17 | ± 1.20 | 529.07 | ± 15.04 |
| MAA | 0.43 | ± 0.02 | 57.39 | ± 1.81 | 827.98 | ± 24.37 | 45.38 | ± 3.93 | 93.88 | ± 4.33 | 508.53 | ± 35.29 |
| PHE | 0.43 | ± 0.02 | 59.76 | ± 2.78 | 804.51 | ± 34.15 | 50.34 | ± 3.35 | 99.92 | ± 2.69 | 504.41 | ± 17.09 |
| ZIN | 0.46 | ± 0.02 | 59.74 | ± 1.78 | 815.17 | ± 39.57 | 50.88 | ± 2.30 | 99.72 | ± 2.56 | 522.74 | ± 17.48 |
| VIT | 0.46 | ± 0.02 | 54.97 | ± 2.06 | 775.05 | ± 32.76 | 44.22 | ± 4.37 | 95.42 | ± 3.16 | 504.38 | ± 18.47 |
| CHI | 0.44 | ± 0.02 | 58.39 | ± 4.13 | 795.25 | ± 48.97 | 47.28 | ± 3.40 | 95.03 | ± 4.22 | 497.75 | ± 23.47 |
| SIL | 0.44 | ± 0.01 | 59.38 | ± 1.48 | 812.14 | ± 10.87 | 51.04 | ± 3.18 | 97.49 | ± 3.00 | 507.48 | ± 12.14 |
| Significance | *** | | ns | | ** | | ns | | *** | | *** | |
|  |  |  |  |  |  |  |  |  |  |  |  |  |
| Year |  |  |  |  |  |  |  |  |  |  |  |  |
| 2016 | 0.48 | ± 0.01 | 56.95 | ± 2.23 | 748.45 | ± 23.55 | 42.60 | ± 2.02 | 94.82 | ± 2.20 | 501.30 | ± 21.55 |
| 2017 | 0.40 | ± 0.02 | 58.45 | ± 2.72 | 830.32 | ± 31.06 | 50.91 | ± 3.83 | 93.97 | ± 4.22 | 484.26 | ± 24.93 |
| Significance | *** | | ns | | *** | | *** | | ns | | ns | |
|  |  |  |  |  |  |  |  |  |  |  |  |  |
| T x Y | ns | | ** | | ** | | ns | | *** | | *** | |

^1^: Means ± S.E.

Values followed by asterisk indicate significant differences between a single treatment group and control group, according to Dunnett’s test (n=4). *******P<0.001; ******P<0.01; *****P<0.05, ns: not significant.

Table S3. Micro-nutrients content of apple skin as affected by treatments with biostimulants and growth season

|  | B (mg/kg DW) | | Fe (mg/kg DW) | | Mn (mg/kg DW) | | Cu (mg/kg DW) | | Zn (mg/kg DW) | | Na (mg/kg DW) | | Si (mg/kg DW) | |
| --- | --- | --- | --- | --- | --- | --- | --- | --- | --- | --- | --- | --- | --- | --- |
| Treatment |  |  |  |  |  |  |  |  |  |  |  |  |  |  |
| CON | 15.93 | ± 0.94 | 29.07 | ± 4.63 | 5.32 | ± 0.41 | 18.27 | ± 3.69 | 3.34 | ± 0.23 | 1.65 | ± 0.13 | 69.60 | ± 3.73 |
| HAL | 16.76 | ± 0.45 | 34.66 | ± 6.68 | 5.00 | ± 0.32 | 19.18 | ± 3.34 | 3.37 | ± 0.07 | 1.50 | ± 0.33 | 74.96 | ± 4.07 |
| APH | 15.80 | ± 0.83 | 26.65 | ± 3.63 | 4.21 | ± 0.34 * | 21.13 | ± 3.75 | 3.31 | ± 0.32 | 1.75 | ± 0.29 | 69.67 | ± 5.38 |
| SEA | 17.15 | ± 0.63 | 33.63 | ± 1.34 | 4.79 | ± 0.25 | 22.40 | ± 2.52 | 3.86 | ± 0.35 | 1.35 | ± 0.26 | 74.48 | ± 3.70 |
| SPI | 16.42 | ± 0.71 | 33.90 | ± 0.84 | 5.36 | ± 0.17 | 20.74 | ± 3.06 | 3.82 | ± 0.23 | 1.30 | ± 0.31 | 72.42 | ± 6.21 |
| MAA | 15.93 | ± 0.90 | 30.67 | ± 3.09 | 5.59 | ± 0.33 | 16.69 | ± 3.03 | 3.81 | ± 0.29 | 1.44 | ± 0.30 | 70.23 | ± 5.31 |
| PHE | 17.89 | ± 0.52 | 32.03 | ± 1.06 | 5.48 | ± 0.32 | 19.27 | ± 2.84 | 3.41 | ± 0.16 | 1.70 | ± 0.32 | 71.71 | ± 3.57 |
| ZIN | 17.47 | ± 0.37 | 33.57 | ± 1.62 | 5.95 | ± 0.35 | 18.83 | ± 2.72 | 4.85 | ± 0.31 *** | 1.67 | ± 0.29 | 71.38 | ± 5.11 |
| VIT | 16.38 | ± 0.70 | 32.72 | ± 1.69 | 5.51 | ± 0.17 | 17.42 | ± 2.71 | 3.50 | ± 0.16 | 1.68 | ± 0.00 | 69.13 | ± 4.36 |
| CHI | 16.09 | ± 0.37 | 30.83 | ± 1.57 | 5.10 | ± 0.18 | 18.93 | ± 2.63 | 3.52 | ± 0.32 | 1.69 | ± 0.18 | 60.18 | ± 2.66 |
| SIL | 16.82 | ± 0.88 | 37.87 | ± 2.55 | 5.33 | ± 0.20 | 18.83 | ± 2.09 | 3.98 | ± 0.23 | 1.20 | ± 0.19 | 71.50 | ± 8.23 |
| Significance | ns | | ns | | *** | | ns | | *** | | ns | | ns | |
|  |  |  |  |  |  |  |  |  |  |  |  |  |  |  |
| Year |  |  |  |  |  |  |  |  |  |  |  |  |  |  |
| 2016 | 17.49 | ± 0.62 | 36.18 | ± 2.96 | 5.45 | ± 0.22 | 25.76 | ± 2.06 | 4.01 | ± 0.24 | 0.45 | ± 0.23 | 78.72 | ± 4.30 |
| 2017 | 15.72 | ± 0.62 | 28.47 | ± 2.63 | 5.03 | ± 0.38 | 12.73 | ± 1.21 | 3.40 | ± 0.29 | 1.91 | ± 0.12 | 62.24 | ± 3.47 |
| Significance | *** | | *** | | ** | | *** | | *** | | *** | | *** | |
|  |  |  |  |  |  |  |  |  |  |  |  |  |  |  |
| T x Y | ns | | *** | | *** | | ns | | ns | | ns | | ns | |

^1^: Means ± S.E.

Values followed by asterisk indicate significant differences between a single treatment group and control group, according to Dunnett’s test (n=4). *******P<0.001; ******P<0.01; *****P<0.05, ns: not significant.
